# Analysis of Usual Consumption of Vitamin D Among Adult Individuals in Italy

**DOI:** 10.3390/nu16234194

**Published:** 2024-12-04

**Authors:** Ranuccio Nuti, Luigi Gennari, Guido Cavati, Carla Caffarelli, Bruno Frediani, Stefano Gonnelli, Antonino Catalano, Cristiano Maria Francucci, Concetta Laurentaci, Giulia Letizia Mauro, Nazzarena Malavolta, Maurizio Mazzantini, Giovanni Minisola, Raffaella Russo, Pasquale Sabatino, Monica Pinto, Sergio Salomone, Luciano Tei, Fabio Vescini, Anastasia Xourafa, Alessandra Cartocci, Sofia Lo Conte, Daniela Merlotti

**Affiliations:** 1 Department of Medicine, Surgery and Neurosciences, University of Siena, 53100 Siena, Italy; ranuccio.nuti@unisi.it (R.N.); luigi.gennari@unisi.it (L.G.); guido.cavati@student.unisi.it (G.C.); carla.caffarelli@unisi.it (C.C.); bruno.frediani@unisi.it (B.F.); stefano.gonnelli@unisi.it (S.G.); alessandra.cartocci@unisi.it (A.C.); 2 Department of Clinical and Experimental Medicine, University of Messina, 98121 Messina, Italy; antonino.catalano@unime.it; 3 Istituto Nazionale di Ricovero e Cura per Anziani Istituti di Ricovero e Cura a Carattere Scientifico, 60124 Ancona, Italy; francuccicm@gmail.com; 4 Azienda Sanitaria Matera, 75100 Matera, Italy; lautitti@gmail.com; 5 Dipartimento delle Discipline Chirurgiche, Oncologiche e Stomatologiche, University of Palermo, 90121 Palermo, Italy; giulia.letiziamauro@unipa.it; 6 Casa di Cura Madre Fortunata Toniolo, 40010 Bologna, Italy; nazzarena.malavolta@gmail.com; 7 Azienda Ospedaliera Universitaria Pisana, 56121 Pisa, Italy; mmazzant@int.med.unipi.it; 8 Ospedale San Camillo, 00152 Rome, Italy; gminisola@scamilloforlanini.rm.it; 9 Department of Medical and Surgical Science, University Magna Grecia, 88100 Catanzaro, Italy; raffaella.russo.md@gmail.com; 10 ASL Salerno, 84121 Salerno, Italy; dott.sabatino@libero.it; 11 Istituto Nazionale Tumori IRCCS Fondazione G. Pascale, 80138 Napoli, Italy; m.pinto@istitutotumori.na.it; 12 Centro Polispecialistico l’Emiro, 90121 Palermo, Italy; sergio.salomone28@gmail.com; 13 Italian Study Group on Metabolic Bone Disorders (GISMO), 00132 Rome, Italy; luciano.tei@libero.it; 14 Azienda Ospedaliera Universitaria Santa Maria della Misericordia, 33100 Udine, Italy; fabio.vescini@asufc.sanita.fvg.it; 15 Azienda Ospedaliera Universitaria Policlinico “G. Rodolico—San Marco”, 95100 Catania, Italy; axourafa@gmail.com; 16 Department of Medical Sciences, Azienda Ospedaliera Universitaria Senese, 53100 Siena, Italy; sofia.loconte@student.unisi.it

**Keywords:** vitamin D, vitamin D deficiency, nutrition

## Abstract

**Background:** The condition of vitamin D (25OHD) deficiency represents an important public health problem. In Europe, hypovitaminosis is common not only in the elderly population but also between 50 and 70 years, both in males and females. Data regarding vitamin D intake in the Italian population are very limited. In a recent paper, reporting data collected by a specific Frequency Food Questionnaire (FFQ), we observed in a small group of healthy subjects that the dietary consumption of vitamin D, both in females and males, was far below the average. **Methods:** With the aim of expanding our preliminary data, we conducted a survey on a large cohort of subjects from different areas of Northern, Central, and Southern Italy. The FFQ contained 11 different questions regarding the amount and type of intake of foods containing ergocalciferol and cholecalciferol. It was submitted to 870 subjects, 627 females and 243 males, with an age range from 40 to 80 years; 31.6% of the studied population was apparently in good health, while 68.4% were affected by different pathologies. **Results:** The present data confirm previous observations: the global quantity of vitamin D intake in 14 days was 70.8 μg (±1.8 SE, ±54.4 SD) in females and 87.5 μg (±1.9 SE, ±57.1 SD) in males; the mean daily intake of vitamin D in females and males was 5.05 μg (±0.5 SE, ±3.8 SD) and 6.25 μg (±0.21 SE, ±4.1 SD), respectively. In healthy subjects, a gradual decrease was observed in the overall intake of vitamin D in both females and males according to an increase in age bracket, ranging from 74.5 μg and 103.8 μg in the 40–50 age group to 54.5 μg and 87.8 μg in the 71–80 age group, respectively. **Conclusions:** In conclusion, the present data, collected in a large Italian cohort, underscore that the daily intake of vitamin D is far below the recommended daily average, thereby contributing to the development of potential hypovitaminosis.

## 1. Introduction

Vitamin D plays an essential role in regulating the metabolism of calcium and phosphate. It promotes intestinal calcium absorption and skeletal mineralization, ensures bone growth, and contributes to bone remodeling [1,2,3,4]. Thus, a correct 25OHD status has a crucial role for preventing metabolic skeletal disorders such as osteoporosis and osteomalacia, and it helps preventing hypocalcemic tetany.

The primary target organ of vitamin D is the intestine, where, through the mediation of calcium-binding protein, it enhances the intestinal calcium absorption. Moreover, vitamin D is involved in inflammatory processes, cellular growth, and the modulation of immune function [5]. In fact, many tissues are also characterized by the presence of vitamin D receptors [6,7].

There are two sources that ensure physiological levels of 25OHD in the blood: sunlight exposure (promoting the production of vitamin D by the skin) and dietary intake. It is believed that cutaneous synthesis accounts for at least 80% of the requirement, while the remaining 20% is obtained through dietary intake [8,9,10]. Cutaneous synthesis involves the production of pre-vitamin D3 from dehydrocholesterol; subsequently, through a temperature-dependent rearrangement of the triene structure, vitamin D3 (cholecalciferol), tachysterol, and lumisterol were synthesized [7,8]. The dermic synthesis of vitamin D3 is influenced by various factors including sunlight and UVB exposure (with a maximal effective wavelength between 290 and 310 nanometers), skin pigmentation, the intensity and duration of sun exposure, and skin aging. Prolonged sun exposure does not lead to toxic levels of D3 because of the photo-conversion mechanism of pre-vitamin D3 into biologically inactive metabolites lumisterol and tachysterol. As is known, sun exposure stimulates the production of melanin, representing an additional mechanism to limit excessive cholecalciferol synthesis [10,11]. Regarding dietary vitamin D intake, vitamin D is present in foods as both D2 (ergocalciferol) and D3 (cholecalciferol). Both forms are absorbed in the small intestine through a mechanism of passive diffusion and active transport proteins across the intestinal membrane [12]. In general, vitamin D is found in limited amounts in foods. The main sources of vitamin D include mushrooms exposed to ultraviolet light and fish such as trout, tuna, and salmon, which have the highest natural amount of cholecalciferol, likely derived from the high content of vitamin D3 in planktonic micro algae at the base of the food chain [13,14,15]. On the other hand, beef liver, chicken, pork, egg yolks, cheese, and milk contain reduced amounts of vitamin D, primarily as cholecalciferol and its metabolite 25OHD3. Foods like fruits, vegetables, rice, and pasta do not contain vitamin D [16].

The condition of 25OHD deficiency undoubtedly represents a significant public health problem [17,18,19,20,21,22,23]. Numerous studies have reported that vitamin D insufficiency is widespread in pregnant women, obese individuals, and subjects who, for various reasons, cannot regularly expose themselves to sunlight [24,25,26]. In particular, in the United States, where cereals and milk are fortified with cholecalciferol, the presence of vitamin D deficiency in the pediatric population is attributed to reduced milk intake, albeit the use of protective creams during sun exposure and the increasing prevalence of obesity may also concur [27]. In Italy, food items are not mandatorily fortified with vitamin D and thus the problem of vitamin D deficiency is still of major relevance, particularly in the winter season when due to the latitude, sunlight exposure does not allow an adequate amount of vitamin D to be synthesized.

Indeed, a high prevalence of 25OHD deficiency has also been documented in Europe [20,28,29], not only in the elderly population but also in the age group between 50 and 70 years, both in males and females [30]. A study conducted in nine European countries revealed that approximately 79.6% of women had 25OHD levels below 80 nmol/L, and 32.1% had levels below 50 nmol/L [31]. Furthermore, another study conducted in 11 European countries demonstrated that 25OHD levels were lower in winter, with values below 30 nmol/L in at least 50% of the population [32]. To date, data regarding vitamin D ingestion in the Italian population are very limited and poorly applicable to subjects at high risk of vitamin D deficiency, such as aging individuals or those with chronic debilitating disorders. Estimates on the prevalence of severe deficiency, deficiency, and insufficiency of vitamin D in Italy were also indirectly achieved by means of multiple-choice questions relating the factors affecting the production, intake, absorption, and metabolism of vitamin D [33]. On the other hand, developing a food questionnaire for the quantitative assessment of vitamin D-containing foods is not straightforward and requires a correct approach in choosing the investigative methodology. Inizio moduloFine modulo.

In a recent paper reporting data collected by a Food Frequency Questionnaire (FFQ) specifically designed to assess dietary vitamin D intake, we observed that in a small group of healthy subjects residing in Central Italy, in both genders, the current dietary ingestion of vitamin D was significantly below the recommended daily allowance (RDA) [34]. In order to expand these preliminary data and with the aim to evaluate to what extent food may contribute to vitamin D status, we used our FFQ to conduct a survey on a large, representative cohort of the Italian population.

## 2. Materials and Methods

The study was carried out using the recently published 14-day FFQ data collection form [34]. The FFQ included questions about the following items: personal data of subjects such as age (subdivided in decades from 40 to 80 years), sex, occupation, educational degree, region of origin, specific eating habits like vegetarian or vegan, and the presence of osteoporosis, cardiovascular, respiratory, kidney, gastrointestinal, endocrine, neurological, and neoplastic diseases. In particular, the FFQ contained 11 different questions regarding the type and quantity of foods containing vitamin D consumed in the 14 days preceding the interview. The following foods were included in FFQ: milk (whole, reduced fat, nonfat, with added vitamin D); corn flakes with vitamin D; yogurt (whole, reduced fat, nonfat, with added vitamin D); cheese (camembert, mozzarella, fontina, gruyere, parmesan, provolone, pecorino, ricotta); meat (beef, pork, fowl, chicken, turkey, lamb); fish, such as bluefish, mackerel, dogfish, flounder, hake, pollock, salmon, salted cod, sea bass, sardine, sea bream, stock fish, swordfish, trout, tuna (differentiated when necessary into fresh, frozen, dry heat, canned); egg (whole, raw, yolk, cooked); food with egg (flan, fried, omelet, meatballs, mayonnaise, pasta); cured meat (baked ham, raw ham, salami, mortadella, bresaola); desserts containing egg, milk, or yogurt (cake, biscuits, ice cream, pastry); and mushrooms. As regards meat and fish, the content in vitamin D was considered as raw food. The FFQ also provided the possibility to indicate a specific food item not included in the list of foods described above. Concerning the frequency of food intake, the question asked how many times that food was consumed in 14 days, from 1 to 14 times. If the daily dose of a specific food was more than the maximum value reported in the questionnaire, it was indicated to spread the amount over more than one day. Subjects included in the present survey were community-dwelling individuals recruited by Clinical Centers of GISMO (Italian Group for the Study of Bone Diseases) and GIBIS (Italian Group for the Study of Bisphosphonates) operating in different areas of Northern, Central, and Southern Italy.

All recruited subjects were properly informed about the study’s goal, and they signed an informed consent form. The USDA National Nutrient Database and the CREA database (“Consiglio per la Ricerca in agricoltura e l’analisi dell’Economia Agraria”) were utilized to calculate the vitamin D amount for each food [35,36]: data coming from foods fortified with vitamin D were excluded. The only exclusion criteria were ages < 40 and >80 years. The survey received approval from the Regional Ethics Committee (Regione Toscana, Sezione Area Vasta Sud Est). The study was conducted from May 2023 to December 2023. Statistical Analysis. All analyses were performed using Statistica 10 (Statsoft, Tulsa, OK, USA) and SPSS (SPSS, version 21.0, IBM Corp., Armonk, NY, USA). Data were summarized as means ± standard errors (SEs) and means ± standard deviation (SD), and *p* < 0.05 was accepted as the value of significance. Probability density functions were also estimated to evaluate the distribution of global and daily vitamin D intake, subdivided by gender and decade of age. Analysis of variance (ANOVA) was used to evaluate the variation in vitamin D intake and different variables including the personal data of the subjects (e.g., age decade, sex, and possible specific eating habits such as vegetarian or vegan) and the presence or absence of pathological conditions. Assuming a margin of error of 5% and a confidence level of 95%, the recruited sample of 870 subjects was considered statistically adequate for the purpose of the study.

## 3. Results

A total of 870 subjects were included, comprising 627 females and 243 males, aged 40–80 years. Table 1 shows the population included in the study, divided into age decades, for both males and females, with the most represented decade being between 61 and 70 years. Most Italian regions were represented in the study, with the exclusion of Valle d’Aosta and Molise. The most represented were Sicily, Campania, Tuscany, and Basilicata, accounting, respectively, for 20.7%, 12.6%, 11.4%, and 10.6% of the total. In terms of education and employment, 39.7% of the subjects had a university degree, 36% had a high school diploma, while 16.2% and 8.2% had a secondary/middle school and primary/elementary school diploma, respectively; 32.5% were retired, while 24%, 13.2%, 12.8%, and 11.4%, respectively, were employees, freelancers, artisans, and housewives.

Overall, 31.6% of the studied population was apparently in good health without the presence of ongoing pathologies, while 68.4% were affected by different disorders, of which osteoporosis and cardiovascular diseases were the most represented (50.1% and 41.3%, respectively). Only 3.4% of the population included in the present survey reported specific dietary habits, of which 3.0% and 0.4% followed vegetarian and vegan habits, respectively. In Table 2 are reported the percentages of user subjects for each food category along with the number of days of intake per 14 days. Milk was among the commonly consumed foods for a longer number of days (56% of cases reported a regular daily intake for all 14 days); conversely, fish, albeit consumed by a large proportion of subjects, was among the less consumed foods per 14 days (1–2 days every 14 days for up to 82.5% of cases).

**Table 2:** Percentages of user subjects for each food category along with the number of days of intake per 14 days.

Food	Users (%)	Daily Frequency of Intakes in 14 Days (% of Subjects)													
		1	2	3	4	5	6	7	8	9	10	11	12	13	14
Milk	63.60%	3.4%	3.6%	1.2%	6.8%	1.1%	5.1%	7.7%	3.8%	0.6%	3.9%	0.2%	2.8%	0.9%	56.4%
Cream	16.30%	53.4%	28.7%	2.8%	7.2%	0.5%	1.0%	/	/	/	/	/	/	/	1.0%
Yogurt	58.60%	5.1%	15.4%	4.7%	24.5%	6.6%	9.5%	9.3%	3.3%	0.4%	5.0%	0.4%	1.5%	/	13.6%
Cheese	93.60%	18.9%	29.5%	10.1%	15.8%	5.7%	6.3%	3%	2.9%	0.4%	3.8%	0.1%	0.7%	0.2%	3.7%
Meat	95.30%	21.9%	40.5%	10.2%	16.4%	4.7%	3.2%	1.0%	0.7%	/	0.3%	/	0.1%	/	0.4%
Fish	88.70%	45.8%	36.7%	6.3%	6.7%	3.1%	2.2%	0.1%	0.2%	/	/	/	/	/	/
Egg	89.00%	33%	41.5%	5.7%	13.7%	1.7%	2.3%	0.5%	0.9%	/	0.2%	0.1%	/	0.1%	0.6%
Food prepared with egg	79.40%	50.5%	34.5%	7.0%	5.5%	1.6%	0.6%	0.3%	0.6%	/	/	/	0.4%	5.7%	1.6%
Cold cuts	80.90%	38.5%	38.1%	9.5%	8.8%	1.8%	1.4%	0.6%	0.6%	/	0.4%	/	0.1%	/	0.3%
Dessert with egg/milk/yogurt	85.10%	15.5%	22.7%	8.6%	11.7%	5.3%	4.1%	3.1%	3.0%	0.4%	4.4%	0.4%	0.5%	0.7%	17.7%
Mushrooms	41.30%	56.6%	27.5%	6.2%	6.7%	1.4%	0.5%	0.5%	0.3%	/	0.6%	/	/	/	/

Each food category was subsequently divided according to its specific food characteristics, as shown in Table 3A,B.

As regards milk, the table indicates that partially skimmed milk was consumed to a higher percentage (51.9%), followed by whole milk (26.4%); for yogurt, whole yogurt was consumed to a higher percentage (33.4%), followed by partially skimmed yogurt (20.8%); for cheese, parmesan was consumed to a higher percentage (23.1%), followed by mozzarella with whole milk (15.2%) and ricotta (13.2%); for meat, chicken was the most consumed (32.7%), followed by beef (17.9%) and pork (16.2%); for fish, canned natural tuna (11.8%), cod (10.5%), and oil-canned fish (7.4%) were that most commonly used.

By correlating the amount of each food consumed over the 14-day period with its vitamin D content per 100 mg, it was possible to calculate the quantity of vitamin D ingested through food in 2 weeks. The global amount of vitamin D intake in 14 days was 70.8 μg (±1.8 SE, ±54.4 SD) [2832 IU (±87 SE, ±2175 SD)] in females and 87.5 μg (±1.9 SE, ±57.1 SD) [3502 IU (±146 SE, ±2287 SD)] in males, with a statistically significant difference between sexes (*p* < 0.001). These data corresponded to a daily intake of 5.05 μg (±0.5 SE, ±3.8 SD) [202 IU (±6.2 SE, ±155 SD)] and 6.25 μg (±0.25 SE, ±4.1 SD) [250 IU (±10.4 SE, ±163 SD)] for females and males, respectively.

A statistically significant difference was observed concerning dietary vitamin D in relation to educational level, with a mean daily intake of 4.1 μg (±0.4 SE) [162 IU (±15 SE)], 5.1 μg (±0.3 SE) [202 IU (±13 SE)], 5.6 μg (±0.2 SE) [224 IU (±10 SE)], and 5.6 μg (±0.2 SE) [225 IU (±8 SE)], respectively, in subjects with a primary/elementary school diploma, middle/secondary school diploma, high school diploma, and university degree (*p* = 0.01, ANOVA). Conversely, daily vitamin D intake did not significantly differ in relation to employment.

**Figure 1:**
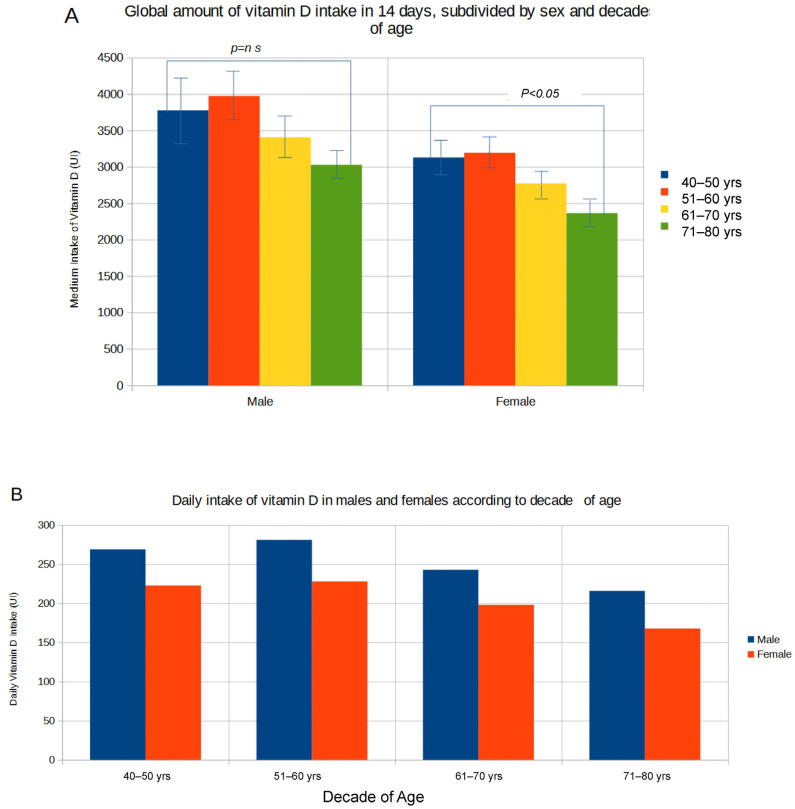
Global amount of vitamin D intake in 14 days (**A**) and mean daily intake of vitamin D in males and females according to age (**B**).

**Figure 2:**
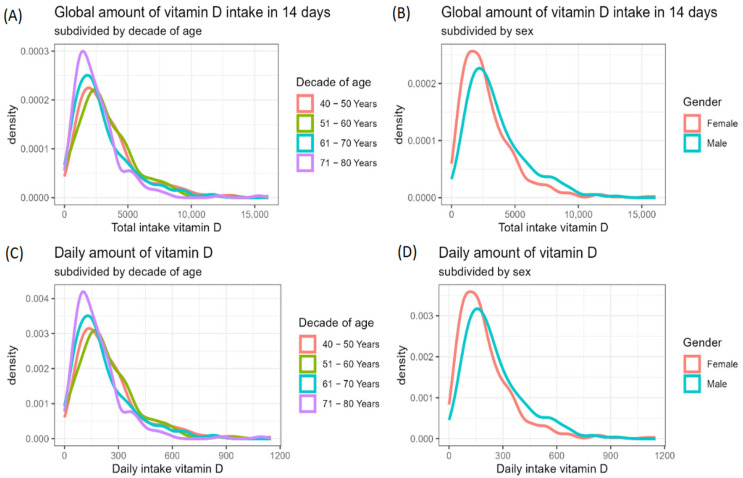
Distribution of global vitamin D intake in 14 days (**A**,**B**) and daily vitamin D intake (**C**,**D**) subdivided by decade of age and sex.

**Figure 3:**
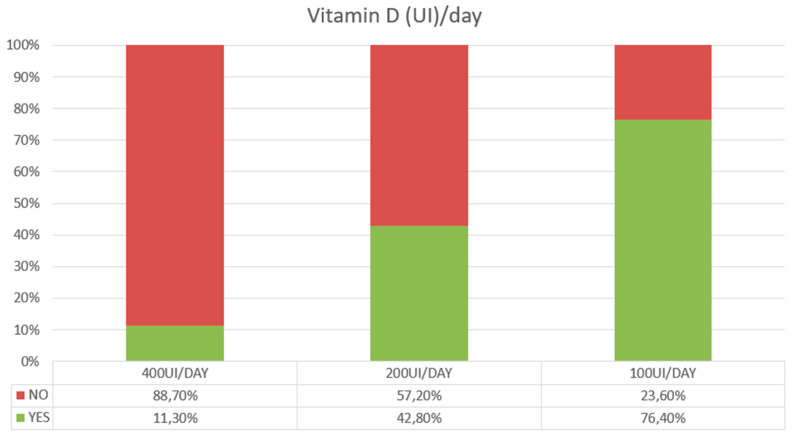
Percentage of subjects reaching 2.5 μg (100 IU), 5.0 μg (200 IU), or 10.0 μg (400 IU) daily ingestion of vitamin D.

Figure 1A shows the amount of global vitamin D intake in 14 days subdivided by sex and decade of ages. With increasing age, a gradual and statistically significant difference (*p* < 0.01) was appreciated as regards the decrease in dietary vitamin D intake in females from values of 78.2 μg (±5.6 SE) [3128 IU (±224 SE)] in the 40–49 age group to 59.1 μg (±4.1 SE) [2362 IU (±162 SE)] in the 71–80 age group; in males, vitamin D intake decreased from values of 94.3 μg (±10.7 SE) [3774 IU (±428 SE)] in the 40–49 age group to 75.7 μg (±4.7 SE) [3029 IU (±187 SE)] in the 71–80 age group. The mean daily amount of vitamin D divided by sex and decade of age is reported in Figure 1B. In both sexes, the daily vitamin D ingestion was very low, ranging from 6.7 μg (±0.7 SE) [269 IU (±30 SE)] in the 40–49 age group to 5.4 μg (±0.3 SE) [216 IU (±13 SE)] in the 71–80 age group in males, and from 5.6 μg (±0.4 SE) [223 IU (±16 SE)] in the 40–49 age group to 4.2 μg (±0.3 SE) [168 IU (±11 SE)] in the 71–80 age group in females. Figure 2 shows the distribution of data subdivided by decade of age and sex: although in both sexes vitamin D consumption was low, it was found to be particularly reduced in the female group; moreover, it tended to decrease with advancing age. Based on these estimates, 76.4% of subjects showed a low vitamin D intake of 100 UI/day or below, with only 11.3% reaching a daily intake of 400 UI/day or above (Figure 3). This latter estimate dropped to 6.8% in subjects over 70 years of age. Subdividing the data of vitamin D intake according to the Italian macro areas of north, center, and south, the mean daily intake was, respectively, 4.9 μg (±0.3 SE) [197 IU (±11 SE)], 5.4 μg (±0.4 SE) [216 IU (±18 SE)], and 5.6 μg (±0.2 SE) [222 IU (±36 SE)], and did not significantly differ (*p* < 0.17).

**Figure 4:**
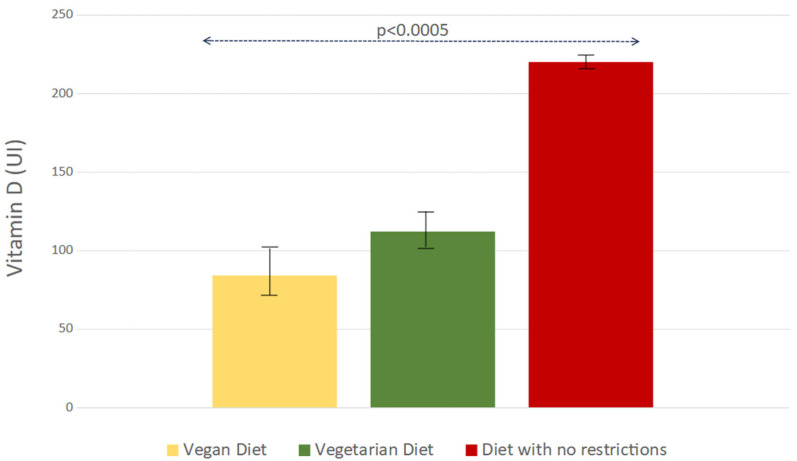
Mean daily vitamin D intake in vegan, vegetarian, and no-restriction diets.

As shown in Table 4, the greatest contribution of vitamin D intake during the 14 days of the survey was supplied by fish, which was found to be remarkably higher than that provided by milk, yogurt, cheese, and meat all together. The overall daily quantity of vitamin D intake was lower in the 595 subjects with one or more comorbidity than in the 275 individuals apparently in good health: 70.8 μg (±1.8 SE [2832 IU ± 73.5 SE]) and 85.5 μg (±1.9 SE [3419 IU ± 77.8 SE]), respectively (*p* < 0.001). The lowest consumption of vitamin D occurred in patients with osteoporosis (66.6 μg ± 1.7 SE [2666 IU ± 71 SE]) or with oncological disorders (66.3 μg ± 1.7 SE [2653 IU ± 71 SE]). Moreover, as shown in Figure 4, the mean dietary vitamin D consumption was lower in vegan and vegetarian subjects than in patients without any particular dietary regimen: 2.1 μg (±0.7 SE) [84 IU ± 31 SE] vs. 2.8 μg (±0.6 SE) [112 IU ± 23 SE] vs. 5.5 μg (±0.1 SE) [220 IU ± 5 SE] (*p* < 0.0005).

## 4. Discussion

The present study confirms our preliminary observation obtained from a limited number of subjects [34], showing that, in the Italian population, the amount of vitamin D intake from foods is limited, with a mean daily intake in females and males of 5.1 μg [202 IU] and 6.2 μg [250 IU], respectively. These data clearly indicate that the average daily vitamin D intake in Italy is very far from the recommended values of 15 μg (600 UI) and 20 μg (800 UI) for subjects aged 51–70 years or above 70 years [13,17], respectively. Moreover, the large standard deviation of the means indicates that the data of vitamin D consumption are spread out, ranging, as regards daily intake, from 47 IU to 357 IU and from 87 IU to 413 IU for females and males, respectively. This aspect, confirmed by the distribution of data evaluated by probability density function, clearly indicates that, in particular conditions, the consumption of foods containing vitamin D is extraordinarily low. This observation suggests that, at least in the winter season, sunlight exposure might not achieve an adequate vitamin D status in a consistent portion of individuals. Such a low intake was a prerogative of both genders and decreased significantly (*p* < 0.001) with aging in females. Fish was undoubtedly the most important and prevalent source of vitamin D, despite the fact that, during the period of 14 days, it was consumed on average only 1–2 times per week.

Importantly, vitamin D intakes were reduced in subjects following vegetarian or vegan eating habits. Moreover, a statistically significant difference was found between the healthy group and patients with pathological conditions, with low values of vitamin D intake in patients with osteoporosis and oncological disorders. Generally, 25OHD status is mainly ensured by adequate sunlight exposure, even if it is not always possible due to various factors such as latitude, skin photo-type, regular outdoor physical activity, and the habitual use of sunscreens. In Italy, adequate sunlight exposure cannot always be guaranteed, especially during the winter season, mainly due to the latitude [37]. Moreover, darker skin subjects require a larger UV dose for the same change in 25OHD, and small, regular UV doses seem to be more efficient for vitamin D synthesis with respect to larger sub-erythemal doses [38].

Whereas a low dietary intake of vitamin D may seemingly play a marginal role in the development of vitamin D status in particular geographical areas, it plays a determining role in conditions where the dermic synthesis of vitamin D is compromised or reduced, thus explaining the widespread diffusion of hypovitaminosis D. For example, in Morocco, characterized by a dry-summer subtropical climate and theoretically optimal sunlight exposure, a high prevalence of hypovitaminosis (vitamin D levels < 30 ng/mL) was described, affecting 90% of the general female population [39]. This was supposed to arise as the consequence of moderate sunlight exposure (41.4% of study participants), but also of a low dietary intake, since 90.78% and 84.21% of participants had a vitamin D intake below the recommended diet allowance (RDA) [39]. This is consistent with our results and further highlights the difficulty of achieving adequate vitamin D status through diet and sun exposure in particular geographic areas and in certain categories of subjects. However, many other factors contribute to hypovitaminosis D: physiological factors such as dark skin pigmentation, pregnancy, and age; pathological conditions such as obesity, malabsorptive syndromes, and hepatic/renal failure; medication; and low ambient UVR level due to a high latitude location.

Indeed, low vitamin D status is recognized as a global health problem [17]. In this regard, the limited consumption of foods containing relevant quantities of vitamin D or fortified foods, the scarce use of vitamin D supplements, lactose intolerance, and socioeconomic status must be considered. In Italy, hypovitaminosis D is common, as confirmed by studies in the general population and patients with metabolic bone disorders [40,41,42,43]. The clinical consequences of vitamin D deficiency may involve bone and muscle health, with increased risks of secondary hyperparathyroidism, fragility fractures, and osteomalacia, and an enhanced risk of falls, particularly in elderly subjects [44,45,46]. Moreover, potential extra-skeletal actions [47,48] suggest vitamin D’s roles in cancer, cardiovascular health, obesity, immunity diabetes, and metabolic syndrome, albeit controlled studies and metanalyses have often yielded unclear and conflicting results [45,49,50,51,52]. The present study is the first extensive survey performed in Italy on adult subjects that underscores the role of insufficient dietary vitamin D intake as a likely explanation for the high prevalence of vitamin D deficiency.

However, our survey retains some limitations, mainly concerning the representativeness of the study at the population level, its cross-sectional design, the lack of a previous criterion-related validation study which uses dietary records, the use of the FFQ instead of a 24 h recall to collect data about food intake, the use of different sources of food composition data even if much of the information was derived by the USDA National Nutrient Database, and the lack of information about the vitamin D status of subjects included in the study.

Overall, our data align with similar observations collected in different countries where total vitamin D intake varied from 3 µg (120 IU) to 5.9 µg (236 IU) per day [53,54], being significantly lower than the RDA suggested by the USA Institute of Medicine [55]. In the US population (2011–2014 and trends from 2003 to 2014), the mean consumption of vitamin D from food and beverage sources was 3.5 μg (140 IU) [56], and the Canadian Health Measures Survey reports a mean intake of vitamin D of 5.1 (204 IU) and 4.2 (168 IU) µg/day for males and females, respectively [57]; in both estimates, the contribution from foods fortified with vitamin D or dietary supplements containing vitamin D were not considered. Low dietary ingestion, with mean values of 3.1 (124 IU) and 2.5 µg/d (100 IU) in adult men and women, respectively, were also observed in the UK National Diet and Nutrition Survey [58]. In Sweden, using a web-based dietary record to assess dietary intake, most participants reported a vitamin D intake below the average requirement (AR), and only 16% of children and 33% of adults met the AR (7.5 μg, corresponding to 300 IU) [29]. In European countries, a mean vitamin D intake well above 5 µg/day (200 IU) was reported mainly in Northern Europe, probably related to the use of fortified foods, while in the other European countries, especially in the Mediterranean area, the consumption is often below 4 µg/day (160 IU) [59]. This report included data from the Italian population obtained from the third Italian national food consumption survey (INRAN-SCAI 2005–2006), even though the study used a generalized 3-day food consumption record and was not specifically designed to assess dietary vitamin D intake [60]. In that study, the mean amounts of vitamin D intake from foods were even lower than those observed in our study and varied between 1.8 and 2.6 µg/d in relation to age and gender. Such a lower intake might have clinical consequences, irrespective of the potential contribution of sunlight exposure on 25OHD status, as observed in two separate studies performed in populations from the Mediterranean area, showing an implication of reduced dietary vitamin D intake on cognitive impairment and cardiovascular disease [46,61].

The opportunity for fortification strategies is suggested in most countries as a public health policy to meet dietary vitamin D recommendations [62], while vitamin D supplements are generally indicated as the recommended alternative when dietary intake is insufficient, in cases of reduced or insufficient UVB radiation, with recommendations for daily intake to be increased to at least 10 μg per day and 25 μg in the elderly (400 IU and 1000 IU, respectively) [63]. Recently, the ESCEO (European Society for Clinical and Economic Aspects of Osteoporosis, Osteoarthritis and Musculoskeletal Diseases) working group recommended 25 μg daily (1000 IU) in patients at an increased risk of vitamin D deficiency [64]. The Italian population predominantly follows a dietary regimen known as the “Mediterranean diet”, a nutritional model inspired by the traditional eating habits of countries bordering the Mediterranean Sea. Studies conducted since the 1950s have consistently emphasized its positive impact on health when combined with a healthy lifestyle. The predominant consumption of plant-based foods such as grains and derivatives, legumes, fruits, vegetables, and extra virgin olive oil has been shown to positively alter the impact of atherosclerotic diseases compared to diets high in red meats and saturated fats. However, it is evident that this dietary pattern often lacks meals with a high content of vitamin D, such as fish, milk, cheese, and eggs. From this perspective, the establishment of an educational health policy aimed at promoting a vitamin D-rich diet including mainly fish, milk, and its derivatives from early childhood to adulthood is believed to be extremely beneficial. This study suggests the implementation of educational healthcare policies, starting from childhood and continuing into adulthood, to promote a correct dietary approach rich in vitamin D [65]. When dietary changes are not feasible, the fortification of certain foods (such as milk and cheese) with vitamin D is advisable [66,67,68]. In cases where these measures are insufficient, vitamin D supplementation, especially in at-risk groups like children, pregnant women, and the elderly, remains a crucial strategy to minimize the risk of hypovitaminosis D and its clinical consequences [69,70]. SIOMMMS (Italian Society for Osteoporosis, Mineral Metabolism and Bone Disease) guidelines indicate the population/condition at risk of hypovitaminosis D and suggest in subjects with hypovitaminosis D a dose of cholecalciferol supplementation between 800 and 2000 IU/day; when serum 25(OH) is <10 ng/mL, an initial loading dose of cholecalciferol 3000–10,000 IU/day (average 5000 IU/day) for 1–2 months is recommended, followed by a maintenance dose of 2000 IU/day [71].

## 5. Conclusions

In conclusion, the results of the present study indicate that the amount of vitamin D intake from food in adult Italian people is markedly low and may contribute to the development of metabolic bone diseases.

## Figures and Tables

**Table 1:** Population included into the study, subdivided by sex and decade of age.

Age Brackets	Females		Males	
	n.	%	n.	%
40–50 years	77	12.3	46	18.9
51–60 years	170	27.1	57	23.5
61–70 years	223	35.6	75	30.9
71–80 years	157	25.0	65	26.7

**Table 3:** (**A**) Percentage of intake for milk, milk-based products, eggs, and derivatives subdivided by item. (**B**) Percentage of intake for meat, fish, cured meats, and mushrooms subdivided by item.

**(A)**		
**Food Group**	**Percentage of Intake for Each Item of Food**	**Global Intake (%)**
Milk	Whole 26.4%; Nonfat 21.7%; Low-fat 51.9%.	63.6%
Cream	For cooking 56.4%; For dessert 43.6%.	16.3%
Yogurt	Whole 33.4%; Nonfat 16.7%; Low-fat 20.8%; Greek nonfat vanilla 16.6%; Parfait 1.5%; Greek fruit 11.9%.	58.6%
Cheese	Gruyere 5.5%; Fontina 2.8%; Parmesan 23.1%; Pecorino 8.3%; Provolone 6.7%; Camembert 0.5%; Gorgonzola 4.2%; Mozzarella whole 15.2%; Mozzarella low-fat 5.8%; Ricotta 13.2%; Cheddar 0.5%; Feta 2.5%; Spread 9.8%; Brie 1.7%; Brick 0.1%.	93.6%
Egg (one egg)	Raw 9.4%; Boiled 31.9%; Fried 24.9%; Omelet 18.9%; Scrambled 14.8%.	89%
Food prepared with egg (more than one egg)	Mayonnaise 7.9%; Flan 5%; Pasta 20.8%; Frittata 25.2%; Meatballs 18.4%; Meatloaf 7.7%; Meat fried 6.9%; Fish fried 6.6%; Stracciatella soup 1.6%.	79.4%
Dessert	Egg and milk 28.6%; Egg 6.1%; Milk 5.6%; Ice cream 25.5%; Cookies 34%.	85.1%
**(B)**		
**Food Group**	**Percentage of Intake for Each Item of Food**	**Global Intake (%)**
Meat	Beef 38.9%; Pork 16.2%; Chicken 32.7%; Guinea fowl 0.4%; Turkey 9.6%; Lamb 2.2%.	95.3%
Fish	Bluefish: anchovy fresh-frozen 7.6%, canned 1.5%; sardines fresh-frozen 1.9%, canned 0.4%; Mackerel fresh 2.9%, canned 1.1%; Sea bass 9.0%; Red snapper 1.4%; Codfish 10.5%; Salt cod 4.3%; Stock fish 1.1%; Hake 2.5%; Guilt-head bream 8.6%; Dogfish 0.5%; Flounder 1.9%; Croaker 0.3%; Sole 1.9%; Eel 0.0%; Salmon: fresh 7.0%, smoked 5.2%, canned 0.4%; Swordfish 5.3%; Tuna: fresh 3.8%, canned natural 11.8%, canned in oil 2.9%; Amber jack 0.4%; Trout 0.6%; Fish eggs 0.1%; Bream 0.0%; Herring: fresh 0.0%, canned 0.1%; Shrimp 4.9%.	88.7%
Cured meat	Raw ham 30.8%; Baked ham 20.8%; Roast ham 2.6%; Salami 10.3%; Bresaola 15.4%; Mortadella 11.6%; Speck 8.4%.	80.9%
Mushrooms	Chanterelles 19.3%; Others 84.7%.	41.3%

**Table 4:** Mean global vitamin D intake in 14 days, subdivided by principal foods.

Food	Mean Vitamin D Intake (UI) ± SE	Users (%)
**MILK**	111.2 ± 9.7	63.6% (553/870)
**YOGURT**	46.1 ± 4.2	58.6% (510/870)
**CHEESE**	150.7 ± 3.5	96.3% (838/870)
**MEAT**	74.3 ± 2.8	95.3% (829/870)
**FISH**	2039.0 ± 64.7	88.7% (772/870)

## Data Availability

The data presented in this study are available on request from the corresponding author. The data are not publicly available due to privacy.
